# Leaching of Bastnasite Ore in a Type IV Deep Eutectic Solvent (EG–FeCl_3_): Taguchi Optimization and Mechanistic Insight by FTIR Evaluation

**DOI:** 10.1021/acsomega.6c01038

**Published:** 2026-07-01

**Authors:** S. Samet Kaplan, M. Seref Sonmez

**Affiliations:** † Metallurgical and Materials Engineering Department, Faculty of Chemical and Metallurgical Engineering, 52971 Istanbul Technical University, 34469 Maslak, Istanbul, Türkiye; ‡ Metallurgical and Materials Engineering Department, Faculty of Engineering and Natural Sciences, Hitit University, 19030 Çorum, Türkiye

## Abstract

The extraction of rare earth elements (REE) from complex mineral matrices typically relies on hydrometallurgical routes involving strong inorganic acids, which pose significant environmental and operational challenges, including silica gel formation. In this study, a sustainable leaching approach was developed for the recovery of Ce, La, and Nd from calcined bastnasite ore using a nonaqueous type IV deep eutectic solvents (DES) composed of ethylene glycol (EG) and iron­(III) chloride (FeCl_3_). A systematic optimization was conducted using the Taguchi L32 orthogonal array design to evaluate the effects of temperature, solid-to-liquid (S/L) ratio, FeCl_3_ concentration, leaching time, and stirring speed. Statistical analysis and stepwise regression modeling identified temperature as the most critical parameter governing extraction efficiency, with the regression models exhibiting high predictive accuracy (*R* ^2^ > 0.87). Molarity and S/L ratio did not exhibit significant effects individually; however, their efficacy became pronounced in conjunction with temperature. The highest total light REE (LREE) extraction efficiency was found to be 62.8% under the conditions of 50 °C, 1 M FeCl_3_, S/L ratio of 0.05 g/mL, and 16 h. Furthermore, the dissolution mechanism was elucidated using FTIR spectroscopy, which provided direct evidence of an in-situ acidification process. Spectral analysis confirmed that ferric ions coordinate with ethylene glycol to form iron-glycolate complexes, releasing protons that drive the dissolution of REE oxides, with water generated as a stoichiometric byproduct. These findings demonstrate that the EG–FeCl_3_ system offers a competitive, simplified, and water-free alternative for processing REE ores, mitigating the limitations of conventional aqueous methods.

## Graphical abstract

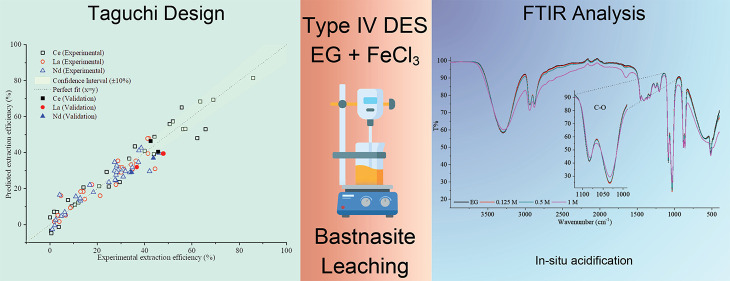

## 1 Introduction

Rare earth elements (REE) are at the heart of modern technological devices and main inputs for renewable energy technologies. From this point of view, their production from both primary and secondary resources is the main challenge for this century. Although extracting REE from secondary resources consumes less energy, it presents distinct challenges such as scrap collection and feedstock heterogeneity.^1–4^ Furthermore, primary resources provide a technically more stable feedstock, despite the associated geopolitical supply risks.^5,6^ For primary extraction, the main processes are beneficiation, calcination, leaching, solvent extraction, and metal reduction.^7^ Within this flowsheet, leaching and solvent extraction are the most water-consuming ones.

In this context, deep eutectic solvents (DES) arise as an environmentally friendly alternative to reagents such as HNO_3_ and H_2_SO_4_ used in these processes.^8^ These solvents possess a unique set of advantageous properties, such as low vapor pressure, nonflammability, low toxicity, ease of preparation, and highly tunable physicochemical characteristics depending on their composition.^9^ The dissolution mechanism of metal oxides in these solvents is primarily driven by the proton-donating capability of the hydrogen bond donor (HBD).^10^ Literature suggests that the acidic nature of the O–H groups facilitates dissolution via a Bro̷nsted acid-type mechanism where the hydroxyl protons interact with the oxide lattice to promote metal extraction.^11,12^ Although different types of DES were utilized for metal leaching in the literature, type III DES based on ethylene glycol (EG) and/or choline chloride (ChCl) are the most widely used for several leaching studies from different sources like coal fly ash,^13,14^ e-wastes,^15–17^ mining residue,^18–20^ chalcopyrite,^21,22^ and Li-ion battery scraps.^23,24^

Type IV DES, formed by combining a metal salt with a HBD, are primarily characterized by their strong Lewis acidic nature. In some literature, this type IV DES is called as metal-based DES (MDES); however, their defining feature remains their ability to facilitate metal extraction through simplified coordination chemistry.^25–27^ Within this category, several metal chloride systems such as those based on ZnCl_2_, CuCl_2_, and AlCl_3_ have been utilized as leaching agents.^28–30^ Recent studies have fundamentally classified iron chloride-based systems as deep eutectic solvents, highlighting their advantageous physicochemical properties like ease of preparation and room-temperature stability for various applications, including biology, biomass treatment, and polymerization.^31–33^ However, despite their proven efficacy in these disciplines, their application as a leaching agent in metal extraction has received limited attention.^22,34^ Therefore, rather than investigating the fundamental thermodynamic properties to validate their DES classification, this study focuses specifically on exploring the practical application of the EG–FeCl_3_ system as an effective lixiviant for REE extraction.

To date, few studies have reported the application of DES to REE minerals.^35–38^ Here, we present a detailed and systematic evaluation of leaching bastnasite mineral in an EG–FeCl_3_ based type IV DES by using the Taguchi experimental design method. The effect of critical parameters such as temperature, FeCl_3_ concentration, and duration was evaluated to understand their influence on the leaching yield. In addition, the reaction mechanism was investigated using FTIR spectroscopy.

## 2 Experimental Design and Procedure

### 2.1 Materials and Equipment

Analytical-grade chemicals, including ethylene glycol (EG) and hydrous iron­(III) chloride (FeCl_3_·6H_2_O), were purchased from Merck. Ultrapure water obtained from the IKA Smart2Pure equipment was used for all solution preparation and analysis purposes. Chemical analysis was conducted by ICP-MS (Thermo-Scientific X-Series-II) equipment. For chemical bonding analysis, FTIR spectroscopy (PerkinElmer Spectrum Two) was used.

The ore sample used in the study was supplied by Turkish Energy, Nuclear and Mineral Research Agency–Rare Earth Elements Research Institute (TENMAK-NATEN). For leaching experiments, the run-of-mine ore was ground to a particle size of below 25 μm and subsequently calcined at 500 °C for 180 min to effectively decompose the fluorocarbonate matrix to REE oxides. According to the elemental analysis, the calcined ore utilized in this study contains 5.3 wt % Ce, 6.0 wt % La, 0.9 wt % Nd, 0.4 wt % Pr, and 0.05 wt % Sm as the primary LREE, alongside major gangue minerals such as CaF_2_ and Ba_2_SO_4_.^34,39^

The EG–FeCl_3_ solvent was prepared via a direct dissolution method. Briefly, the required molar amount of hydrous iron­(III) chloride was added directly into liquid EG and heated to 50 °C. The mixture was continuously agitated using a magnetic stirrer until the metal salt was completely dissolved, resulting in a homogeneous, clear liquid. The preparation is relatively simple compared to conventional DES synthesis, thereby offering a highly simplified and energy-efficient preparation protocol.

### 2.2 Leaching Experiments and Design

Leaching optimization studies were carried out on the calcined ore obtained under the optimized conditions determined in the previous study.^34^ The experiments were carried out in a three-neck glass reactor immersed in a silicone oil bath to ensure uniform heating, and the solution was mixed with an overhead stirrer. The study followed the Taguchi L32 orthogonal array design to systematically investigate the effects of process parameters on the leaching efficiency. A total of five different parameters were examined, with four factors set at four levels and one factor at two levels. Leaching yield of each REE was calculated based on the mass balance of the solid, and liquid phases, as shown in eq 1:

$$\mathrm{yield}\;(\%)=\frac{C_{L}\times V_{L}}{C_{S}\times m_{S}}\times 100$$ 1

where *C*_L_ is the concentration of the specific REE in the pregnant leachate (mg/L), *V*_L_ is the volume of DES used (L), *C*_S_ is the initial content of the REE in the calcined bastnasite ore (mg/g), and *m*_S_ is the mass of the calcined ore sample (g) added to the reactor.

The parameters selected for the leaching process were the temperature (*X*_1_), solid/liquid ratio (*X*_2_), FeCl_3_ concentration (*X*_3_), leaching time (*X*_4_), and stirring speed (*X*_5_). The experimental factors, their codes, and the corresponding levels are detailed in Table 1.

**Table 1:** Experimental Parameters and Levels Used in the Taguchi L32 Design

parameter	code	level 1	level 2	level 3	level 4
temperature (°C)	*X*_1_	25	50	75	100
solid/liquid ratio (g/mL)	*X*_2_	0.025	0.05	0.1	0.2
FeCl_3_ concentration (M)	*X*_3_	0.125	0.250	0.5	1
time (h)	*X*_4_	2	4	8	16
stirring speed (rpm)	*X*_5_	300	600		

### 2.3 Statistical Analysis

The relationship between the experimental parameters and extraction efficiencies was modeled using stepwise regression analysis, and calculations were conducted with Minitab software. This method constructs regression equations by considering the effect of statistically significant variables based on a specified range of *p*-values.^40^ In this study, the criteria for the stepwise model were set as follows: variables with a *p*-value of less than 0.05 were entered into the model, while those with a *p*-value greater than 0.15 were removed. This approach allowed for the elimination of non-significant variables, resulting in simpler equations with higher determination coefficients (*R* ^2^) that better represent the effect of experimental parameters on the results.

The performance of four main outputs was measured: the extraction efficiencies of Ce, La, Nd, and total LREE (∑LREE). Although the extraction results for Sm and Pr were also monitored, their individual efficiencies were evaluated under the LREE yield rather than separately, as their low concentrations could lead to erroneous interpretations when analyzed individually.

## 3 Results and Discussion

### 3.1 Statistical Analysis and Model Fitting

All experimental and calculated extraction percentages from regression equations are given in the Supporting Information Table, and regression equations are given in Table 2. All *R* ^2^ values are greater than 87%, indicating a strong correlation between the experimental data and the predicted values.

**Table 2:** Regression Equations

	regression equations	*R* ^2^ (%)
Ce	–48.1 + 1.372*X*_1_ + 0.929*X*_4_ – 7.1*X*_3_ + 218.7*X*_2_ – 0.00433*X*_1_^2^ + 0.424*X*_1_ *X*_3_ – 4.504*X*_1_ *X*_2_ + 3.183*X*_4_ *X*_3_ – 9.74*X*_4_ *X*_2_	94.83
La	–24.29 + 1.331*X*_1_ + 4.60*X*_3_ + 27.9*X*_2_ – 0.00787*X*_1_^2^ + 0.313*X*_1_ *X*_3_ – 1.914*X*_1_ *X*_2_	87.97
Nd	–37.45 + 1.384*X*_1_ + 0.365*X*_4_ + 22.29*X*_3_ + 48.8*X*_2_ – 0.0073*X*_1_^2^ – 1.704*X*_1_ *X*_2_	88.26
∑LREE	–33.54 + 1.411*X*_1_ – 0.02*X*_4_ – 1.4*X*_3_ + 78*X*_2_ – 0.00686*X*_1_^2^ + 0.323*X*_1_ *X*_3_ – 2.939*X*_1_ *X*_2_ + 1.694*X*_4_ *X*_3_	93.39

To evaluate the significance of individual factors and their interactions, an analysis of variance (ANOVA) was conducted (Table 3). The analysis reveals that the leaching process is governed by a complex interplay of parameters, with temperature emerging as the most critical factor. Individual effects of the parameters are discussed in the following section.

**Table 3:** Regression Statistical Results (*p* and *F* values)

	**Ce**		**La**		**Nd**		**∑LREE**	
**factor**	***F***	***p***	***F***	***p***	***F***	***p***	***F***	***p***
temperature (*T*)	25.13	0.000	40.86	0.000	52.63	0.000	45.39	0.000
time (*t*)	3.77	0.065			4.91	0.036	0.00	0.946
FeCl_3_ concentration (*M*)	0.29	0.598	0.23	0.635	33.63	0.000	0.02	0.897
solid/liquid ratio (S/L)	10.55	0.004	0.34	0.565	1.18	0.288	2.63	0.118
*T* ^2^	4.92	0.037	28.28	0.000	26.65	0.000	21.25	0.000
*T* × *M*	5.04	0.035	4.66	0.041			4.79	0.039
*T* × S/L	23.29	0.000	6.95	0.014	6.14	0.020	16.19	0.001
*t* × *M*	22.65	0.000					10.57	0.004
*t* × S/L	8.09	0.009						

### 3.2 Mean Value Analysis and Main Effect

The impact of each experimental factor on the leaching yield was evaluated using mean-value analysis, as illustrated in the main-effect plots in Figure 1. This analysis reveals distinct behaviors for each element, which corroborates the ANOVA results.

**Figure 1:**
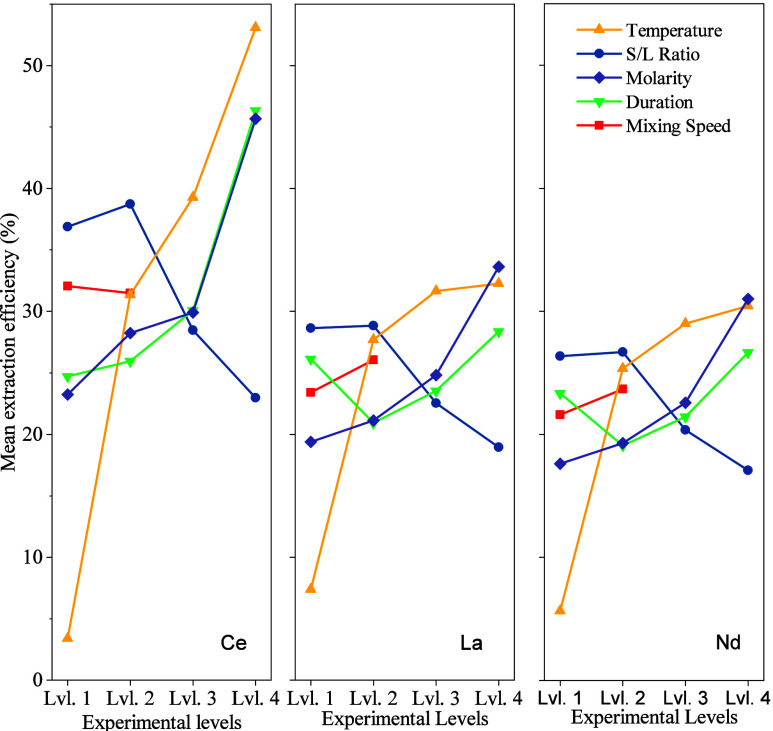
Mean value analysis.

Temperature demonstrated a highly significant effect on the extraction efficiency of all elements (Ce, La, and Nd) and LREE, exhibiting high *F*-values (e.g., *F* = 52.63 for Nd) and a *p*-value of 0.000 across the board. The relationship between temperature and extraction yield is not strictly linear; the significance of the quadratic temperature term (*T* ^2^) suggests that yields increase rapidly and may approach saturation at higher temperatures. For instance, increasing the temperature from 25 to 100 °C resulted in a nearly 7-fold increase in the average Ce extraction and raised the average LREE extraction from 5.5 to 40.4%.

While temperature is universally dominant, the influence of FeCl_3_ concentration varies by element. For Ce and La extraction alone was not statistically significant (*p* > 0.05); however, for Nd, it proved to be a significant parameter (*p* = 0.000), with yields rising notably from 20.7 to 38.1% as FeCl_3_ concentration increased from 0.125 to 1 M. Although concentration was not individually significant for ∑LREE, its interaction with temperature (*T* × *M*) was found to be significant (*p* = 0.039), indicating that the efficacy of Fe^3+^ ions in the dissolution mechanism is thermally dependent.

The S/L ratio showed a significant negative correlation for Ce extraction (*p* = 0.004), where lower ratios favored higher yields. For Nd and ∑LREE, the S/L ratio was not significant in isolation but became highly relevant when analyzed in interaction with temperature (*T* × S/L, *p* ≤ 0.020). This interaction suggests that at higher temperatures, high solid loading may limit dissolution efficiency due to saturation or transfer limitations.

The effect of leaching time was generally secondary compared to temperature. It was marginally significant for Ce (*p* = 0.065) and statistically significant for Nd (*p* = 0.036), particularly showing benefits at extended durations (16 h). For ∑LREE, time was significant primarily through its interaction with concentration, suggesting that longer durations are more effective when reagent availability is high.

Notably, stirring speed was the least influential parameter, showing no statistically significant effect on the extraction of Ce, La, or ∑LREE, and only a negligible increase for Nd. This lack of sensitivity to hydrodynamic conditions suggests that the leaching system is predominantly under chemical reaction control rather than diffusion control.

In conclusion, single-factor optimization is insufficient for this system. The analysis confirms that while temperature is the primary driver, maximizing extraction efficiency requires a multivariate approach that accounts for significant interactions, particularly among temperature, concentration, and solid loading. The parameter effect on leaching efficiency is summarized and given in Table 4.

**Table 4:**
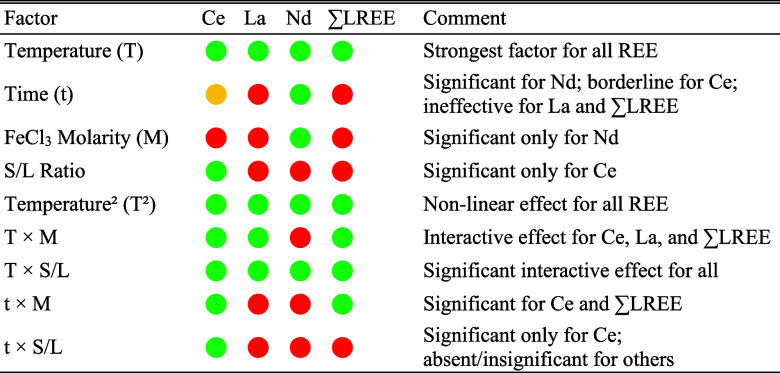
Summary of Parameters’ Influence on Leaching Efficiency

### 3.3 Model Validation Experiments

To evaluate the predictive accuracy of the regression models derived from the statistical analysis, two confirmatory experiments were conducted under random conditions that were distinct from the initial Taguchi orthogonal array. The extraction efficiencies predicted by the developed equations were compared with the actual experimental results to determine the reliability of the model. A comparison of these two experimental results is given in Table 5.

**Table 5:** Comparison of Experimental and Predicted Extraction Yields for the Validation Experiments Conducted at (I) 75 °C, 0.125 M, 8 h, 0.025 S/L and (II) 50 °C, 1 M, 8 h, 0.05 S/L

**exp. no**	**element**	**experimental (%)**	**predicted (%)**	**variation**
I	Ce	40.5	45.7	5.2
	La	31.9	36.7	4.8
	Nd	29.0	34.5	5.5
II	Ce	46.4	42.6	–3.8
	La	39.5	47.8	8.3
	Nd	36.9	43.8	6.9

In the first validation set (75 °C, 8 h, 0.125 M FeCl_3_, 0.025 S/L), the model exhibited a high degree of consistency with the experimental data, albeit with a slight conservative bias. The actual extraction efficiencies for Ce, La, and Nd were consistently higher than the predicted values by margins of 5.2, 4.8, and 5.5, respectively. In the second validation set (50 °C, 8 h, 1 M FeCl_3_, 0.05 S/L), the model displayed reasonable predictive capability. It underestimated the leaching of La and Nd by 8.3 and 6.9, respectively, while slightly overestimating the Ce extraction by −3.8.

The validation experiments conducted to assess the model’s robustness revealed a specific trend. While the model showed reasonable accuracy for cerium (deviations largely within ±10%), it tended to overestimate the extraction efficiencies for La and Nd, with deviations reaching up to 15–20% in some validation points shown in Figure 2. This suggests that while the regression models are effective in mapping the directional influence of parameters and identifying optimum conditions, they should be used as a semiquantitative guide. Final yield determinations for La and Nd specifically require experimental verification due to the model’s optimistic prediction tendency.

**Figure 2:**
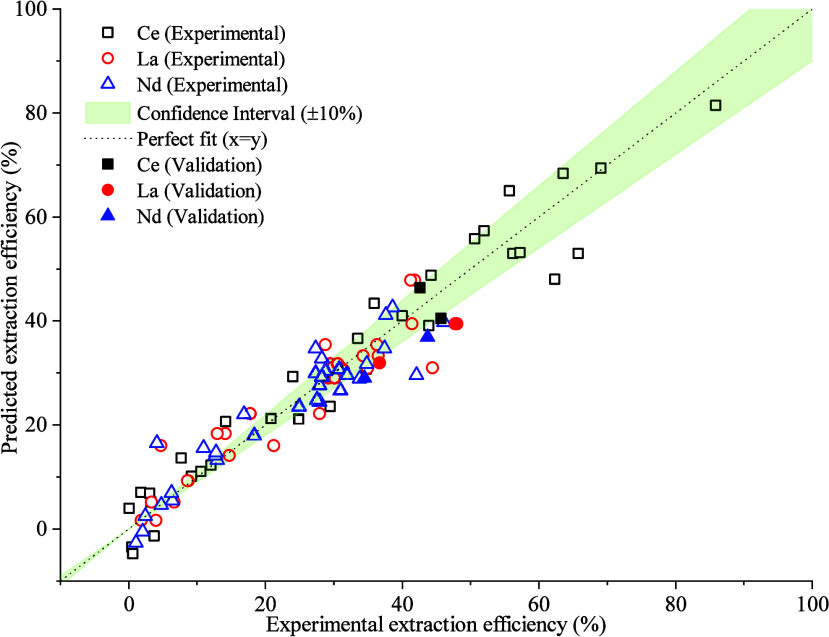
Parity chart for experiments.

To validate the optimal process conditions and test the model’s limits beyond the experimental design space, more experiments were conducted in triplicate at 125 °C, 24 h, 0.125 M FeCl_3_, and a 0.025 S/L ratio. The average experimental extraction efficiencies were obtained as 51.3% (±4.0) for Ce, 31.4% (±2.3) for La, and 30.9% (±2.2) for Nd. When compared with the regression model predictions, Nd showed exceptional agreement with a deviation of only −1.97% (predicted as 28.93%), demonstrating the model’s robustness even outside the primary design range (25–100 °C). However, deviations were observed for Ce (predicted as 78.87%) and La (predicted as 19.30%). Triplicated extreme temperature trials’ results are given in Table 6. The sharp decline in the Ce experimental yield at 125 °C and 24 h points to an element-specific secondary precipitation triggered by prolonged thermal stress. The stability of La and Nd yields under the same conditions strongly suggests that bulk chemical saturation and total solvent degradation are not the limiting factors. While the literature indicates that Ce often exhibits distinct precipitation behaviors in high-temperature aqueous systems,^41–43^ whether a similar element-specific mechanism occurs in this nonaqueous EG–FeCl_3_ system requires detailed future validation. These results confirm that while the model is effective for optimization within the studied parameters, experimental verification remains essential for high-temperature extrapolation, thereby establishing 100 °C as the strict operational upper limit for the system.

**Table 6:** Extreme Temperature Experiments’ Results

element	exp. result (%)	predicted result (%)	variance
Ce	51.3	78.87	+27.57
La	31.4	19.30	–12.10
Nd	30.9	28.93	–1.97

While conventional aqueous acid leaching can achieve REE extractions over 80%, it requires highly concentrated mineral acids and extreme temperatures.^44,45^ In contrast, the proposed EG–FeCl_3_ system achieves a competitive maximum LREE extraction of 62.88% (and up to 85.88% for Ce) at a mild temperature of 50 °C, avoiding the generation of toxic acidic wastewater and eliminating the silica gelation problem associated with traditional methods.^46^

#### 3.3.1 Solvent Regeneration

For the proposed system to be viable as a closed-loop hydrometallurgical process, the recovery of the dissolved LREE and the subsequent regeneration of the lixiviant are essential. As comprehensively evaluated in the recent literature regarding solvent recovery, the successful recycling of a deep eutectic solvent strongly depends on its specific physicochemical properties. Various techniques can be employed, including antisolvent addition, crystallization, membrane filtration, short-path distillation, and liquid–liquid extraction.^47^ Directly regenerating the lixiviant back to its original state via simple thermal or precipitation methods presents unique challenges because of the high boiling point of EG (−197 °C) and the risk of Fe^3+^ coprecipitation, which could strip the structural iron from the solvent matrix. Due to these complexities, solvent extraction (SX) emerges as one of the most fundamental and practically applicable methods for selectively separating target metals and regenerating the lixiviant used in the study. Accordingly, the specific separation of REE from the leachate and the associated regeneration aspects have been systematically addressed via SX in our separate study.^48^

### 3.4 Characterization of the Lixiviant and Proposed Leaching Mechanism

To understand the chemical environment responsible for the dissolution of REE, the structural interactions within the lixiviant at different FeCl_3_ concentrations were investigated using FTIR spectroscopy prior to the leaching process, as given in Figure 3. The spectral analysis reveals changes primarily associated with the hydration of the system and the solvation of iron ions. The O–H stretching band centered at around 3300 cm^–1^ exhibits a broadening trend and a decrease in transmittance as the FeCl_3_ concentration increases. This change is most distinct at the 1.0 M concentration, where the increased population of hydroxyl groups leads to a more extensive hydrogen-bonding network. Concurrently, a characteristic peak emerges around 1650 cm^–1^, corresponding to the H–O–H bending vibration. The intensity of this peak correlates positively with the water coming from the hydrated FeCl_3_ source and its subsequent interaction with the glycol matrix. The interaction between the solvent and iron ions is further evidenced in the fingerprint region, as highlighted in the inset of the graph. The C–O stretching bands between 1000 and 1100 cm^–1^ show a reduction in intensity without a significant shift in wavenumber. Furthermore, spectral alterations observed in the low-frequency region approaching 500 cm^–1^ suggest the presence of metal–oxygen interactions (Fe–O).^49^ These observations indicate that while the molecular skeleton of ethylene glycol remains intact, the solvation of Fe^3+^ ions and H_2_O molecules modifies the dipole environment and vibrational characteristics of the solvent.

**Figure 3:**
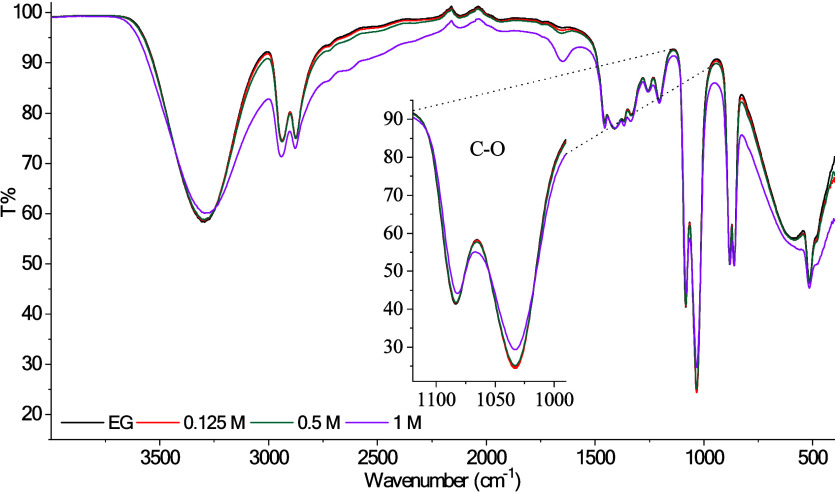
FTIR spectra of EG–FeCl_3_ solvents at different FeCl_3_ molarities.

Figure 4 compares the FTIR spectra of pure EG, the initial lixiviant (0.125 M), and the final pregnant leach solution. The most distinct spectral alteration observed after the leaching process is the intensification of bands associated with water molecules. While the initial lixiviant shows a slight increase in O–H vibrations due to the hydrated iron salt, the leachate spectrum (blue line) exhibits a further and more pronounced broadening in the O–H stretching region (3000–3600 cm^–1^) and a marked increase in the H–O–H bending vibration intensity around 1640 cm^–1^. This accumulation of water in the final solution confirms that water is generated as a stoichiometric byproduct of the leaching reaction.

**Figure 4:**
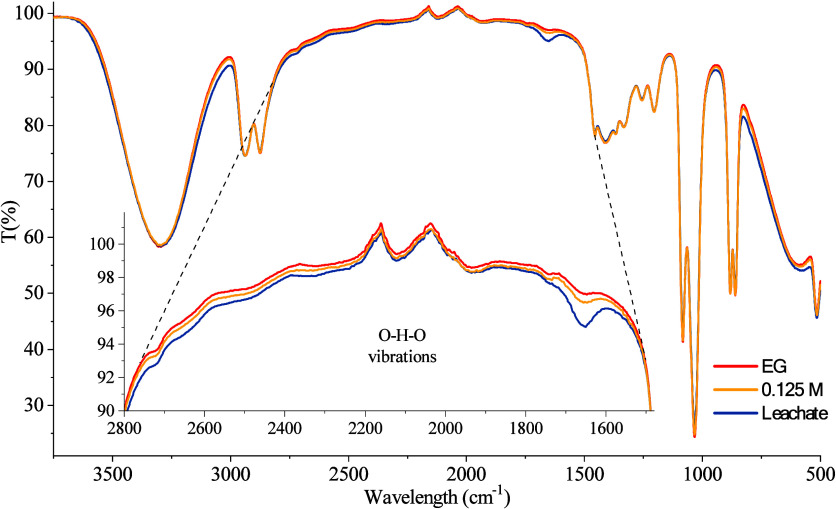
FTIR spectra of EG–FeCl_3_ solvent before and after leaching.

Synthesizing the FTIR findings—specifically the spectral evidence of iron solvation and the stoichiometric generation of water—REE dissolution is proposed to proceed via two sequential reaction stages.

#### 3.4.1 Stage 1: In Situ Acidification (Solvolysis)

As evidenced by the spectral changes in the fingerprint region and O–H stretching bands, ferric ions act as a Lewis acid. They coordinate with EG molecules, weakening the O–H bonds and releasing protons into the solution. This reaction was proposed as the formation of Fe glycolate chelates by creating an Fe octahedron by four oxygen atoms from two EG molecules and two chlorides from FeCl_3_. In this reaction mechanism, a proton is released into the solvent, while one uncoordinated chloride ion is in the outer sphere for charge neutrality. This in situ acidification step is described by eq 2:

$${\mathrm{FeCl}}_{3}+2\mathrm{EG}↔\left[{\mathrm{FeCl}}_{2}\left(\mathrm{EG}\right)\left(\mathrm{EG}-H\right)\right]+{H^{+}}_{\left(\mathrm{solv}.\right)}+{{\mathrm{Cl}}^{-}}_{\left(\mathrm{solv}.\right)}$$ 2

#### 3.4.2 Stage 2: REE Dissolution and Water Generation

The protons generated in the first stage attack the rare earth oxides (REE_2_O_3_) present in the calcined ore. The FTIR analysis of the pregnant leach solution, showing an intensification of the H–O–H bending vibration at 1640 cm^–1^ confirms that water is produced as a stoichiometric byproduct of this dissolution reaction. This step proceeds according to eq 3:

$${\mathrm{REE}}_{2}O_{3}+6{H^{+}}_{\left(\mathrm{solv}.\right)}\to 2{{\mathrm{REE}}^{3+}}_{\left(\mathrm{solv}.\right)}+3H_{2}O$$ 3

Consequently, the extraction mechanism is driven by the synergistic effect of the solvated protons dissolving the oxides and the iron-glycolate complexes preventing the reprecipitation of the dissolved ions.

## 4 Conclusion

This study demonstrates the efficacy of a nonaqueous solvometallurgical leaching route using an ethylene glycol (EG) and iron­(III) chloride system for the extraction of REE from calcined bastnasite ore. Through a systematic Taguchi L32 experimental design, the leaching process was optimized, revealing that temperature is the dominant factor governing extraction efficiency for Ce, La, and Nd, followed by the solid/liquid ratio and FeCl_3_ concentration. Stepwise regression modeling yielded robust predictive equations (*R* ^2^ > 0.87) that were validated within the design range, though severe deviations observed exclusively for Ce at extreme conditions (>100 °C, 24 h) suggest an element-specific precipitation that requires further investigation.

Furthermore, FTIR characterization elucidated the underlying dissolution mechanism, providing critical evidence for an in situ acidification process. Spectral analysis confirmed that Fe^3+^ ions act as strong Lewis acids, coordinating with EG to weaken O–H bonds and release protons (H^+^), which drive the dissolution of REE oxides while generating water as a stoichiometric byproduct. By reducing the direct use of external mineral acids, the EG–FeCl_3_ system demonstrates competitive performance as a type IV DES, achieving a maximum LREE extraction of 62.8% and up to 85.8% for Ce at a moderate temperature of 50 °C. Consequently, this study not only establishes optimized parameters for high-yield REE recovery but also provides a fundamental understanding of the iron-glycolate-mediated solvation chemistry, paving the way for the sustainable processing of complex REE ores.

## Supplementary Material



## Notes

All individual experiments’ measured and calculated leaching extraction efficiency at different experimental conditions (PDF)
