# Supplementary material for: Leaching of Bastnasite Ore in a Type IV Deep Eutectic Solvent (EG–FeCl3): Taguchi Optimization and Mechanistic Insight by FTIR Evaluation
Source: ACS Omega. 2026 Jul 1;11(27):39902–9. doi: 10.1021/acsomega.6c01038 (PMC13382672; doi:10.1021/acsomega.6c01038)
Supplement: Supplementary file 1 [file ao6c01038_si_001.pdf]

Supplementary File

Leaching of bastnasite ore in a type IV deep eutectic solvent (EG-$FeCl_3$): Taguchi optimization and mechanistic insight by FTIR evaluation

S. Samet Kaplan[12,*], M. Seref Sonmez[1]

[1] Metallurgical and Materials Engineering Department, Faculty of Chemical and Metallurgical Engineering, Istanbul Technical University, 34469, Maslak, Istanbul, Türkiye

Table 1 Experimental (Exp.) and Calculated (Calc.) Leaching values of La, Ce, Nd, and total LREE.

Temp. (°C)	Duration (h)	$FeCl_3$ Molarity (M)	Solid Liquid ratio (g/mL)	Mixing Speed (rpm)	Ce (Exp.)	Ce (Calc.)	La (Exp.)	La (Calc.)	Nd (Exp.)	Nd (Calc.)	∑LREE (Exp.)	∑LREE (Calc.)
25	2	0.125	0.2	300	0.11	3.91	1.82	1.63	1.06	-2.66	1.05	-0.43
50	4	0.25	0.2	300	10.55	11.01	14.72	14.09	12.95	13.25	12.62	11.37
75	8	0.5	0.2	300	29.5	23.55	27.91	22.17	24.9	23.55	28.18	23.24
100	16	1	0.2	300	69.13	69.38	36.48	33.31	34.85	31.76	49.38	53.46
25	2	0.25	0.1	300	0.47	-3.52	3.33	5.18	2.03	-0.49	1.99	0.38
50	4	0.125	0.1	300	12.03	12.2	14.16	18.34	12.68	14.11	12.94	15.58
75	8	1	0.1	300	63.59	68.34	41.86	47.78	38.59	42.6	50.40	55.67
100	16	0.5	0.1	300	55.67	65.02	29.46	31.71	28.27	32.77	39.83	46.05
25	4	0.5	0.05	300	3.7	-1.32	8.74	9.28	6.42	5.5	6.34	4.32
50	2	1	0.05	300	34.75	30.7	41.44	39.45	37.42	34.7	38.04	34.51
75	16	0.125	0.05	300	40	41.01	29.22	28.99	27.31	29.96	33.34	32.50
100	8	0.25	0.05	300	56.22	52.94	29.68	30.91	28.8	30.36	40.20	39.12
25	4	1	0.025	300	3.29	5.12	4.67	15.99	4.12	16.49	4.01	10.93
50	2	0.5	0.025	300	24.86	21.12	31.04	31.01	27.92	24.46	27.90	27.17
75	16	0.25	0.025	300	51.99	57.34	28.75	35.39	27.37	34.72	37.87	42.30
100	8	0.125	0.025	300	57.27	53.09	31.42	30.51	30.76	30.62	41.65	38.96
25	16	0.125	0.2	600	0.62	-4.79	4	1.63	2.41	2.45	2.41	2.25
50	8	0.25	0.2	600	9.17	10.11	14.72	14.09	12.79	14.71	12.16	12.99
75	4	0.5	0.2	600	20.82	21.26	17.76	22.17	16.85	22.09	18.69	19.93
100	2	1	0.2	600	43.95	39.09	34.28	33.31	31	26.65	37.83	30.03
25	16	0.25	0.1	600	1.78	6.99	6.64	5.18	4.76	4.62	4.37	6.02
50	8	0.125	0.1	600	7.72	13.61	12.98	18.34	10.94	15.57	10.52	16.34
75	4	1	0.1	600	50.62	55.79	41.23	47.78	37.63	41.14	44.62	48.98
100	2	0.5	0.1	600	35.9	43.37	30.59	31.71	27.91	27.66	32.41	34.47
25	8	0.5	0.05	600	3.05	6.82	8.62	9.28	6.26	6.96	5.99	7.63
50	16	1	0.05	600	85.88	81.45	48.01	39.45	46.15	39.81	62.88	57.95
75	2	0.125	0.05	600	24.05	29.26	30.12	28.99	27.56	24.85	27.17	29.81
100	4	0.25	0.05	600	62.31	47.99	34.92	30.91	33.78	28.9	45.71	37.50
25	8	1	0.025	600	14.18	20.59	21.22	15.99	18.35	17.95	17.80	17.63
50	16	0.5	0.025	600	65.76	52.99	44.44	31.01	42.08	29.57	52.45	38.74
75	2	0.25	0.025	600	33.55	36.6	36.32	35.39	31.93	29.62	34.48	36.65
100	4	0.125	0.025	600	44.26	48.75	31.32	30.51	28.27	29.16	36.14	38.19

[2] Metallurgical and Materials Engineering Department, Faculty of Engineering and Natural Sciences, Hitit University, Çorum, Türkiye
